# Emerging strategies in nanotechnology to treat respiratory tract infections: realizing current trends for future clinical perspectives

**DOI:** 10.1080/10717544.2022.2089294

**Published:** 2022-07-27

**Authors:** Minhua Chen, Zhangxuan Shou, Xue Jin, Yingjun Chen

**Affiliations:** a Emergency & Intensive Care Unit Center, Department of Intensive Care Unit, Zhejiang Provincial People’s Hospital, Affiliated People’s Hospital, Hangzhou Medical College, Hangzhou, China; b Department of Pharmacy, The Second Affiliated Hospital of Zhejiang Chinese Medical University, Hangzhou, China; c Center for Clinical Pharmacy, Cancer Center, Department of Pharmacy, Zhejiang Provincial People’s Hospital (Affiliated People’s Hospital, Hangzhou Medical College), Hangzhou, China; d Department of Infectious Diseases, People’s Hospital of Tiantai County, Taizhou, China

**Keywords:** Respiratory tract infections, bacterial infections, smart nanosystems, nanodiagnostics

## Abstract

A boom in respiratory tract infection cases has inflicted a socio-economic burden on the healthcare system worldwide, especially in developing countries. Limited alternative therapeutic options have posed a major threat to human health. Nanotechnology has brought an immense breakthrough in the pharmaceutical industry in a jiffy. The vast applications of nanotechnology ranging from early diagnosis to treatment strategies are employed for respiratory tract infections. The research avenues explored a multitude of nanosystems for effective drug delivery to the target site and combating the issues laid through multidrug resistance and protective niches of the bacteria. In this review a brief introduction to respiratory diseases and multifaceted barriers imposed by bacterial infections are enlightened. The manuscript reviewed different nanosystems, i.e. liposomes, solid lipid nanoparticles, polymeric nanoparticles, dendrimers, nanogels, and metallic (gold and silver) which enhanced bactericidal effects, prevented biofilm formation, improved mucus penetration, and site-specific delivery. Moreover, most of the nanotechnology-based recent research is in a preclinical and clinical experimental stage and safety assessment is still challenging.

## 1. Introduction

Respiratory infections are the leading cause of mortality and it is estimated that by 2030 the death rate due to respiratory-related diseases would reach 20% of all deaths (Mizgerd, 2006; Troy & Bosco, 2016). The respiratory tract is continuously exposed to microorganisms (bacteria, viruses, and fungi). If pathogenic microorganisms are not cleared from the lungs upon inhalation, it leads to respiratory tract infections. These infections are categorized into upper and lower respiratory tract infections. Lower respiratory tract infections are mainly caused due to bacterial infections which have more deleterious effects and represent the highest death rates in developing countries. Thus, putting a gruesome financial burden on the health care system (Z. Huang et al., 2021). *S. pneumoniae*, *H. influenzae*, *Moraxella catarrhalis, S. aureus, P. aeruginosa, M. tuberculosis, S. pyogenes* are predominant pathogens in respiratory tract infections (Shima et al., 2016). Respiratory tract infections are difficult to treat owing to the localization of microbes that resides deep inside the tract and are embedded with thick protective mucus and biofilms layer (Tucker et al., 2021). Therapeutics can be administered to the target site either systematically or directly through the respiratory tract. A higher dosage of the drug is required in case of intravenous or oral delivery (systemic delivery) to reach the target site (Baranyai et al., 2021). While in the case of pulmonary delivery, the drugs in solution form either have low retention time in the lungs or metabolic enzymes inactivate them, therefore, making the therapeutics ineffective. Though antibiotics like macrolides and quinolone can enter the host cell through diffusion, they are removed from the host cell via an efflux pump (X. Z. Li & Nikaido, 2009; López et al., 2021). Antimicrobial resistance is another major concern in public health which is escalating because of the misuse of drugs. The emergence of ‘superbugs’ has rendered multifaceted challenges for the pharmaceutical industry. Respiratory infection-causing pathogens (*P. aeruginosa*, *M. tuberculosis, S. pneumonia,* and *H. influenza)* exhibit lower susceptibility to the many classes of antibiotics. Thus, the higher demand for antimicrobials further contributes to the development of antimicrobial resistance and aggravates the situation (Guitor & Wright, 2018).

**Figure 1.**
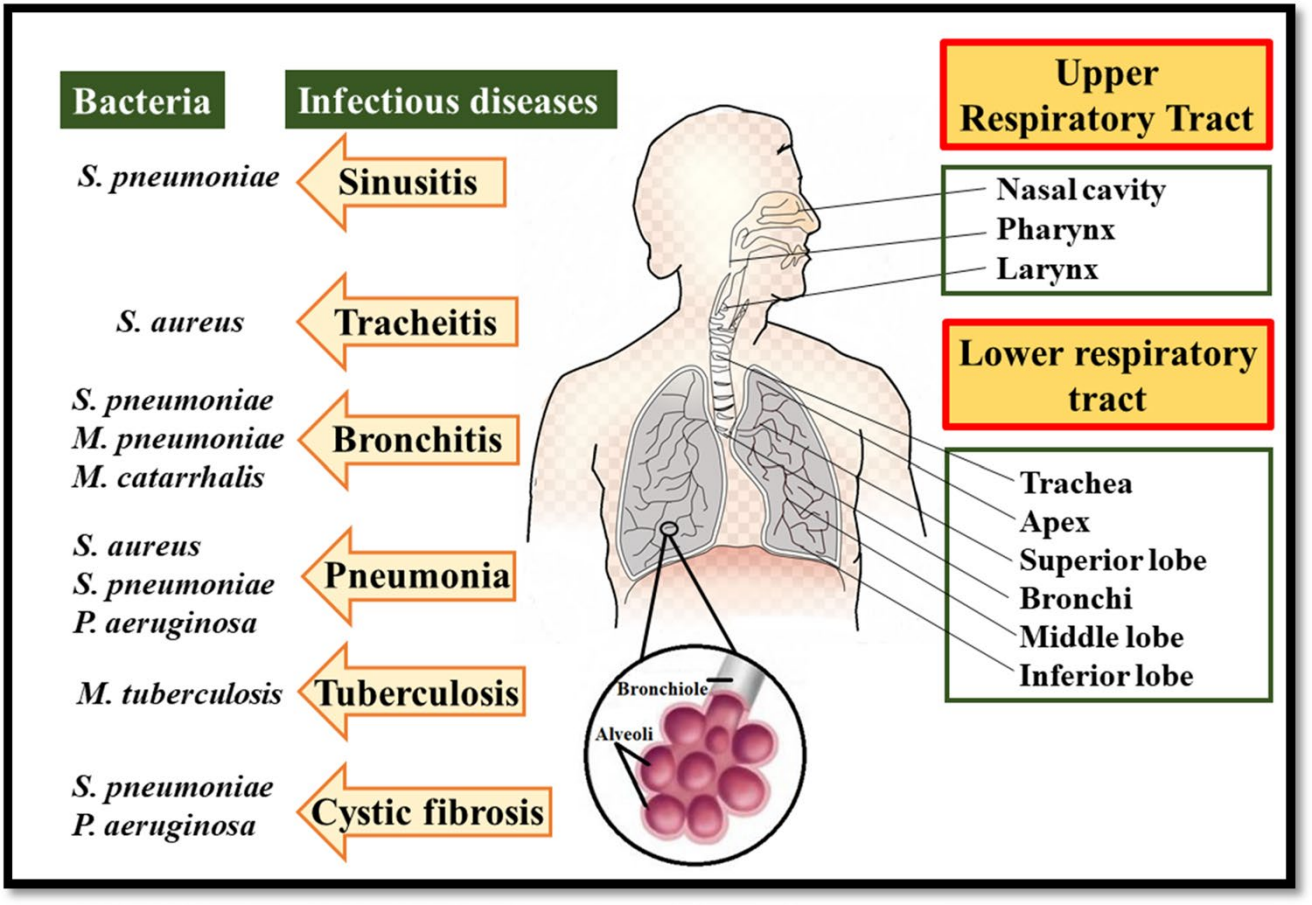
Anatomy of the respiratory tract and its bacterial infection.

The huge cost and strenuous efforts required in the discovery of new antimicrobials have limited their discovery and the focus has been shifted toward the development of effective formulations to deliver the drug at the targeted site. Nanotechnology is one of the most promising alternative approaches to evade the limitations of conventional formulations. Nano-encapsulation of drugs provides various advantages, i.e. protection of antimicrobial drugs in the biological environment and providing sustained drug release with higher retention time (Falciani et al., 2020), enhancement of drug encapsulation and solubility (Ingle et al., 2016), reducing the risk of side effects by specific targeting (Y. Yu et al., 2022), simultaneous delivery of multiple drugs (Lababidi et al., 2020), crossing the barriers, and combating the resistance mechanisms developed by the bacteria (Mullis et al., 2021). Nanoparticles enter the mammalian cells either via phagocytosis or pinocytosis. So the employment of nanosystems can help in targeting the intracellular bacteria (Kamaruzzaman et al., 2017). Another benefit offered by nanotechnology is the uniform distribution of the drug in the lungs. The homogenous size of nano-suspensions ensures the encapsulation of the drug in each droplet in comparison with the microparticulate form of free antibiotics (Ingle et al., 2016) (Figure 1).

## 2. Respiratory infectious diseases

### 2.1. Tuberculosis

Tuberculosis (TB) is caused by *M. tuberculosis* and remains a fatal disease across the globe. *M. tuberculosis* is a facultative intracellular parasite that replicates inside the host after being carried through aerosol droplets. *M. tuberculosis* may affect other organs, i.e. liver, kidney, bones, and the central nervous system, but predominantly it invades the pulmonary tract. When TB extravagates outside the lungs it is known as extra-pulmonary TB (Da Silva et al., 2016; Nasiruddin et al., 2017). *M. tuberculosis* is the most critical pathogen in the global antimicrobial resistance crisis. The first-line drugs used in the treatment of standard TB include four oral antibiotics regimens, i.e. pyrazinamide, isoniazid, rifampin, and ethambutol (Pinheiro et al., 2011). Patients find it difficult to stick to this regime due to prolonged and frequent dosing; unable to penetrate the alveolar macrophages where bacilli reside; instability in gastric acid which leads to the development of drug-resistant strains and increases the risk of treatment failure (Ginsberg & Spigelman, 2007). Bedaquiline was the first drug approved in more than 40 years (Deoghare, 2013). However, reformulation of existing drugs in nanosystems is a possible way for the effective treatment of TB.

### 2.2. Cystic fibrosis

Cystic fibrosis (CF) is a fatal chronic pulmonary infection that occurs due to a defect in transmembrane protein which is known as cystic fibrosis transmembrane conductance regulator (CFTR) which regulates chloride ion secretion (Winstanley et al., 2016). The chloride ion imbalance decreased the volume of surface liquid in the airways, mucus dehydration, and lessens mucus clearance which eventually leads to the secretion and accumulation of viscous mucus in the airways and causes bronchial obstruction (Ong et al., 2019). In the early stages of the disease, *S. aureus* and *H. influenza*e are found in abundance whereas, in the advance stages *P. aeruginosa*, *Burkholderia cenocepacia*, *Stenotrophomonas maltophilia*, and *Achromobacter xylosoxidans* are found profusely (Klinger-Strobel et al., 2015). *P. aeruginosa* infection is mostly accountable for most of the premature deceases of CF patients (De Boeck, 2020). Thus, inhibiting chronic colonization caused by *P. aeruginosa* is the key aim for the early treatment of CF. The lungs of CF patients even after receiving antibiotic regimens and aerosolization of tobramycin are persistently colonized by *P. aeruginosa* (Moreau-Marquis et al., 2008). To improve the chloride ion transport functions, CFTR modulators, i.e. ivacaftor, lumacaftor, and tezecaftor have been used. However, refinement in toxicity, specificity, and adverse effects is required to expand the use and effectiveness of these medicines (Robinson et al., 2018).

### 2.3. Asthma

A chronic inflammatory lung disease that is characterized by reversible airway obstruction and bronchial hyper-responsiveness. The Global Initiative for Asthma (GINA) proposed some guidelines for the management of asthma: alleviate and control symptoms; halt the loss of pulmonary function; reduce asthma exacerbations and minimize adverse effects (Cheng & Chen, 2019). It is found that *S. pneumoniae, H. influenzae, and M. catarrhalis* are associated with asthma exaggerations (Kraft, 2000). Some uncommon microbial pathogens includes; *M. pneumoniae* and *Chlamydia pneumoniae* which induce and exacerbate asthma (Darveaux & Lemanske, 2014). The conventional inhaled anti-asthma drugs have improved bioavailability and efficacy. Glucocorticoid, an inhaled corticosteroid (ICS) is the most effective drug to control airway inflammation caused by asthma. However, ICS might not provide immediate relief and cause long-term side effects such as decreased rate of treatment adherence (Adouni Lawani et al., 2018) which can accelerate the progression of the disease as well as lung remodeling, and leads to pulmonary deterioration (Pascual & Peters, 2005). Moreover, the long-term high dose hormone inhalation will bring harmful effects, i.e. inhibition of the adrenal axis; oral fungal infections; and osteoporosis (L. Wang et al., 2019).

### 2.4. Pneumonia

**Figure 2.**
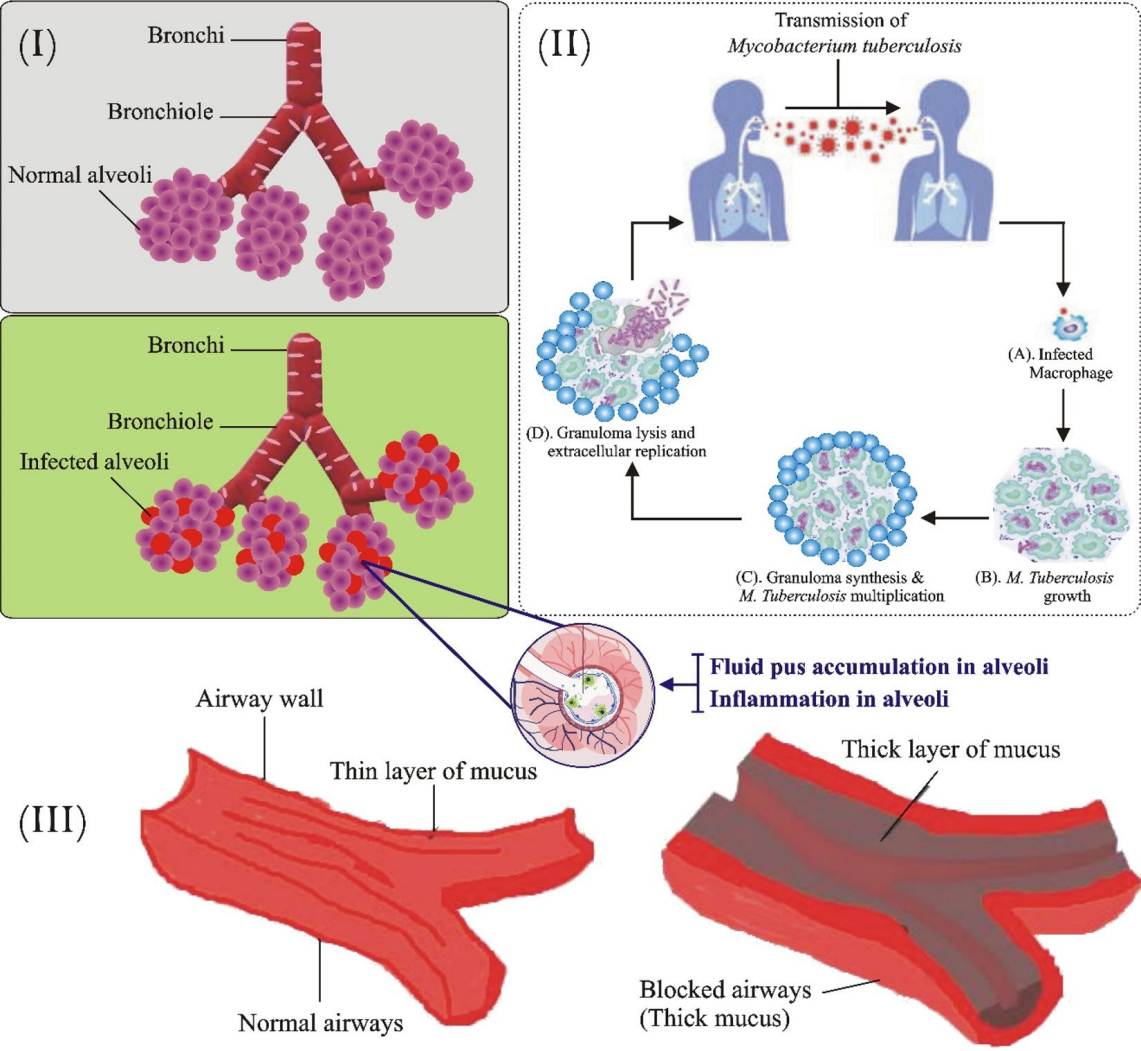
Bacterial respiratory infectious diseases. i. Pathophysiology of pneumonia: Pathogens enter via inhalation and reach lower airways. Alveoli releases cytokines and inflammatory mediators which lead to alveolar fluid accumulation. ii. Pathogenesis of TB: M. tuberculosis enters the respiratory tract through inhalation and infects alveoli. In the first step, alveolar macrophages recognize, engulf, and try to destroy bacilli. In the second step, bacilli start to grow within the infected alveolar macrophages which ultimately transform into granuloma. Most human with infected TB don’t exhibit a progression of the disease and remains in a latent state. However, some infected persons progress to the final stage where cavities are filled with free-floating bacteria and spread in the lungs causing pulmonary TB. iii. Comparison of normal and Cystic fibrosis patient airways- Illustration showing normal and highly viscous mucus airways in lungs and respiratory tubes.

Pneumonia is an inflammatory condition of the lungs which affect primarily lung air sacs (alveoli). Alveolar spaces are occupied by pus and fluid which distress breathing and confine oxygen uptake. The lesions appear inside alveoli, often associated with buds of granulation tissue that inhibit the bronchiolar lumen resulting in lung abrasions (Al-Tubaikh, 2010). Many etiological agents are accountable for pneumonia including pathogenic bacteria; *S. pneumoniae, H. influenzae, S. aureus*, gram-negative bacilli, *M. pneumoniae, Acinetobacter baumanni, Stenotrophomonas maltophilia,* and opportunistic fungi that reach alveoli by micro aspiration of oropharynges secretion (Sanivarapu & Gibson, 2021). Certain viruses, i.e. coronavirus, adenoviruses, influenza virus, and respiratory syncytial viruses are also responsible for the spread of viral pneumonia (Muhammad et al., 2022). The therapy is initiated after the conformation of an etiological agent and the severity of the disease then the treatment starts in a rational way to treat the development of these resistant strains. However, the current treatment of pneumonia is ineffective due to adverse toxic effects related to antibiotics (vancomycin) and inefficient effects against multidrug resistance (B. Kim et al., 2018). Therefore, nanotechnology is an emerging technique to overcome hurdles in this regulatory fatal infection (Figure 2).

## 3 Bio-barriers: Hindrance in the effective delivery of drugs

Many biological barriers in the respiratory tract impede the delivery of drugs at the targeted site. To formulate an effective drug for respiratory tract infections, it is necessary to consider these biological barriers and the obstacles that are imposed by them. The characteristics of these barriers are different in the case of normal and pathophysiological conditions. Thus, emphasizing the need for rational designing of the therapeutics for effective treatment of respiratory tract infections.

### 3.1. Micro-environment of the respiratory tract

A heterogeneous lung lining fluid is distributed continuously throughout the respiratory tract. The trachea, bronchi, and bronchioles (conducting parts) are lined with a mucus gel, while the pulmonary surfactants and alveolar sub-phase fluid line the alveoli (A. W. Ng et al., 2004). Mucus is composed of water, globular proteins, lipids, DNA, mucins, salts, and cellular debris. It acts as a protective layer and helps in lubrication Mucus blocks the passage of pathogens and foreign substances to the underlying epithelium. Mucins are glycoproteins that contribute to the viscoelasticity of the mucus membrane (Zanin et al., 2016). In pathological conditions, the microenvironment of the respiratory tract is affected. The chronic bacterial infections in Cystic fibrosis change the pH of the respiratory tract from almost neutral to acidic. This altered pH induces conformational changes in the structure of mucin protein which can impact the interaction of nanoparticles and mucus (Poschet et al., 2002; F. Wan et al., 2020). Furthermore, respiratory tract diseases lead to excessive production and dehydration of mucus that also disrupt the interactivity of mucus and therapeutics. The production of extremely viscous mucus in a certain pathological environment may lead to embolism in the trachea, bronchi, and bronchioles, thus further obstructing the passage of drugs from the respiratory tract. The low clearance rate and higher accumulation of mucus create room for microbial growth and thus cause infection.

Pulmonary surfactants are lipid-protein complexes that help in the stabilization of the alveoli and also contribute to innate immune defense. The interaction between the nanoparticles and the surfactant can influence the normal functioning of the surfactants. Certain lung diseases are associated with the abnormal composition and functioning of pulmonary surfactants. These studies reflect the need for novel designing of the nanoparticles for respiratory tract infections, considering the altered microenvironment in case of pathological conditions (Veldhuizen & Haagsman, 2000).

### 3.2. Bacterial biofilms

**Figure 3.**
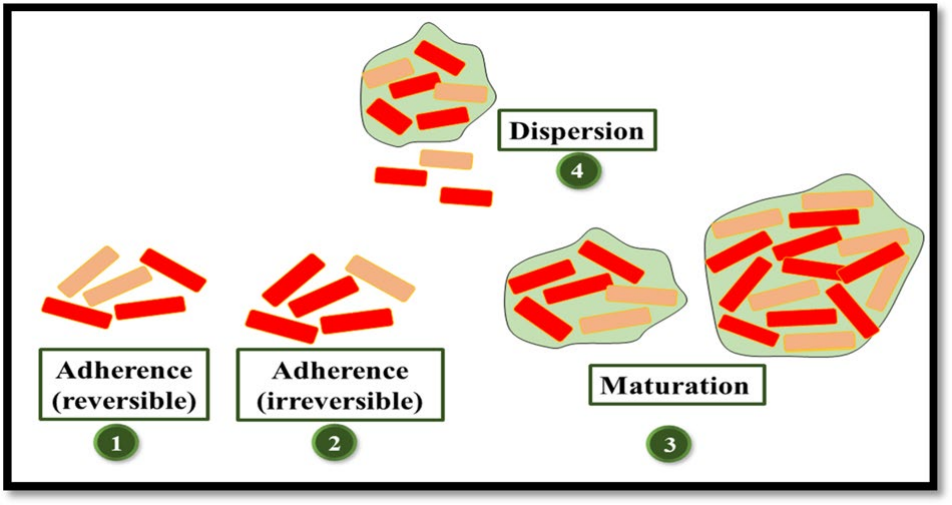
Steps involved in biofilm formation.

Biofilms refer to the closely associated community of microbes inside a network of carbohydrates, proteins, nucleic acids, and other substances known as extracellular polymeric substances (EPS) (Flemming & Wingender, 2010). There are five stages involved in the development of biofilms (Figure 3). The first stage comprises of reversible adherence of microbes to a surface which is followed by the irreversible attachment of microbes and the release of EPS by microbes. In the next stage which is the early stage of biofilm, small colonies of microbes are immersed in the polymeric matrix. Upon maturation of the biofilms, the microcolonies of bacteria are separated through the open water channels. In the last dispersion stage, the biofilm starts the release of planktonic cells and the aggregates of bacteria (Blasi et al., 2016).

Biofilms render antibiotic resistance in bacteria either through the extra-polymeric matrix that acts as a barrier to the entry of antibacterial agents or through the sharing of resistant genes between resistant and susceptible microbes via horizontal gene transfer. Owing to these resistance films it is very difficult to treat bacterial infections. A bacterial biofilm requires 1000 times more concentration of antibiotic than the minimum inhibitory concentration (MIC) of antibiotic needed to kill bacteria (Gnanadhas et al., 2015). Biofilms are primarily involved in lower respiratory tract infections which are one of the major causes of death in developing countries (Sharma et al., 2021). Kolpen et al. (2022) observed the presence of biofilms in the sputum of both acute and chronic lung infection patients, thus indicating the predominance of biofilms formation in respiratory tract infections. *P. aeruginosa* which is most prevalent in the respiratory tract infections in cystic fibrosis patients survives the antibiotic treatment due to its ability to form biofilms (Cipolla et al., 2014; Koch & Hoiby, 1993)

### 3.3. Intracellular bacteria

Various bacteria create a niche inside the host cells from where they can dodge the host immune system and spread to other sites in the body. These bacteria which localize inside a host cell are known as intracellular bacteria. *M. tuberculosis, Salmonella enterica, Chlamydi trachomatis,* and *Listeria monocytogenes* are well-known intracellular bacteria. In addition, some extracellular bacteria can also persist inside the host cell. *S. aureus, Escherichia coli,* and *P. aeruginosa* are the few extracellular bacteria that can be hosted inside the cell (Kamaruzzaman et al., 2017). Despite having various antibacterial therapeutics almost two-thirds of them are considered ineffective to treat the infection caused by intracellular pathogens. It is also expected that microorganisms may still be present inside the host cells even though the *in-vitro* susceptibility tests reveal the opposite (Tucker et al., 2021). Microorganisms may persist in the respiratory tract for a long time even when prescribed antimicrobials have been expected to be active based on conventional *in-vitro* susceptibility testing.

*M. tuberculosis* is an intracellular bacteria that reside inside the macrophages. Bacteria enter the macrophage through complement and mannose receptors. In order to ensure its survival, *M. tuberculosis* alters the lysosome trafficking and phagolysosome fusion. The host cells defend themselves via apoptosis of macrophages that are resisted by bacteria. However, bacteria can block the apoptosis process of macrophages by utilizing TNF-receptor-dependent mechanisms (Gordon & Read, 2002). It is also found that the commensal microorganism which protects against the microbial pathogens at the mucosal surface can also develop infections. These opportunistic bacteria become involved in pathogenicity in case of a compromised immunity of patients, a change in microbiota, or in case of broken skin or mucosal membranes. These microorganisms not only persist but their transmission also went undetected which poses another challenge in the effective treatment and detection of infections (Thakur et al., 2019).

## 4. Nanosystems employed for respiratory tract bacterial infections

### 4.1. Smart nano-systems

**Figure 4.**
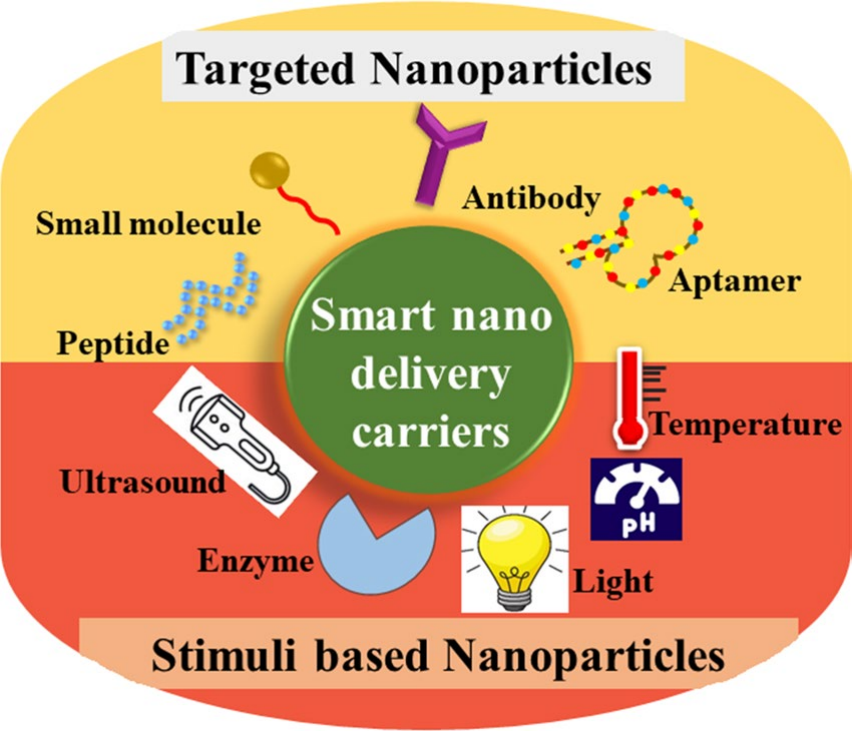
Schematic representation of smart nano delivery carriers.

Nanotechnology offers myriad benefits in the medical field. These benefits can be cashed mainly through the engineering of nanoparticles. The focus is now shifted to the engineering of smart nanoparticles. As the name implies, these nanoparticles are fabricated to ensure the drug delivery to specified target sites while minimizing the side effects. These nanoparticles are designed to be target-specific either through targeted delivery (utilizing the receptor-ligand bonding) or through stimuli-responsive delivery of therapeutics Figure 4.

**Figure 5.**
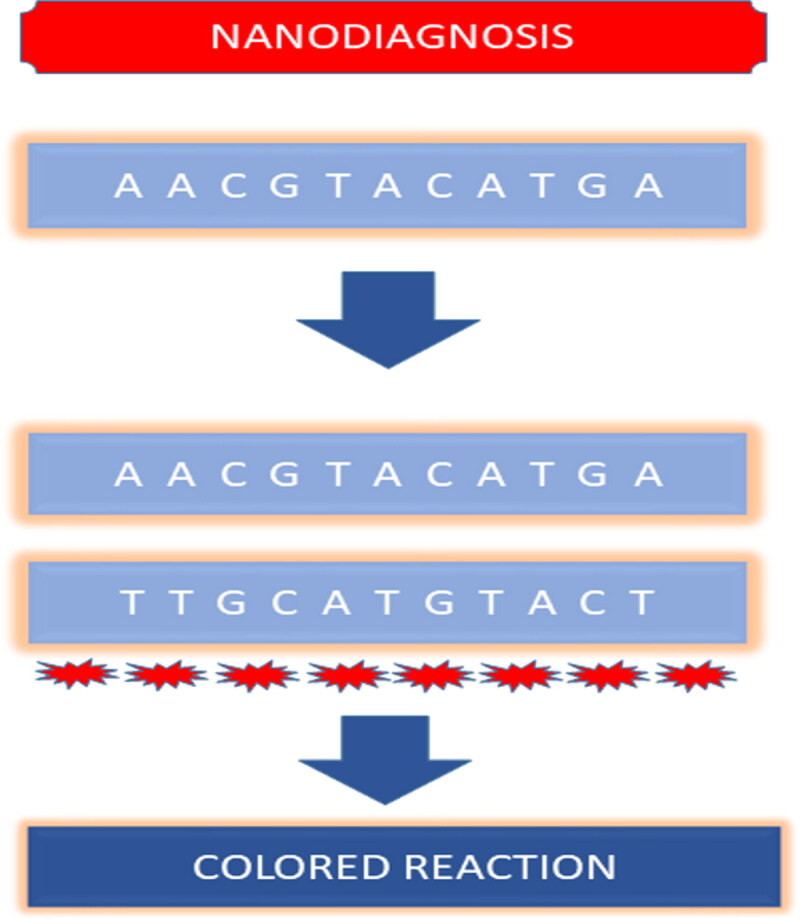
Hybridization-based nano diagnostics for pathogens. (Upon hybridization of target DNA AACGTACATGA with the probe containing nanoparticles the nanoparticles will change their color thus helps in detection of the target DNA sequence).

Baranyai et al., formulated multifunctional nano-systems in which he utilized both targeted delivery of drug and the stimuli-responsive release of drug at the target site. The reactive oxygen species (ROS) responsive, Moxifloxcin encapsulated nano-system of 4-(hydroxymethyl) phenylboronic acid pinacol ester (HPAP)-modified cyclodextrin was prepared for the pulmonary bacterial infections. The phenylboronic acid-modified nano-systems are ROS sensitive and they release the drug when the H_2_O_2_ level reaches an optimum range of (0.5–1.0 mM). Thus, the higher levels of the ROS at the site of infection were availed for the stimulus-responsive release of moxifloxacin. For the targeted delivery of drug to the macrophages and mucus, the surface of nano-systems was functionalized with Polyethylene glycol (PEG) and folic acid (FA). These smart nanosystems exhibited more promising results than the free drug (Moxifloxcin) or the non-targeted nanoparticles on the *P. aeruginosa* infected mouse models. A novel pH-sensitive, surface-modified, and imipenem-loaded silver nanocomposite (IPM@AgNPs-PEG-NOTA) was constructed to evaluate the antibacterial activity in MLE-12 (mouse lung epithelial) cell lines and mice against carbapenem-resistant *A. baumannii*. The results demonstrated efficient antibacterial activity, increased ROS production, and membrane damage which induced oxidative stress, halted cell wall formation, and interfered with metabolic pathways. Additionally, the nanosystem also inhibited the formation of biofilm. *In-vivo* studies showed the secretion of proinflammatory cytokines was restrained and repair of infected tissues in mice was promoted as compared to imipenem. This novel drug delivery system also represented effective bactericidal properties in clinical isolates of *A. baumannii* which paved way for clinical studies (X. Li et al., 2021) (Figure 5).

### 4.2. Liposomes

Liposomes are spherical lipid vesicles mainly composed of phospholipids and cholesterol. This colloidal form is a self-assembled lipid bilayer with an amphiphilic domain, inner hydrophobic core, and outer layer of the lipid bilayer (Bulbake et al., 2017). Liposomes can encapsulate drugs with different solubility either in water core or phospholipid bilayer and enhance the solubility of loaded drugs (Ngan & Asmawi, 2018). Due to the similar composition of the lipid bilayer and cell membrane, the liposome carriers could cross various biological barriers and enhance the absorption and therapeutic effects of encapsulated drugs (Rudokas et al., 2016). Liposomal formulations that contain fusogenic lipid bilayers can interfere with bacterial membrane, thereby allowing greater drug retention and intracellular delivery of entrapped drugs (Sachetelli et al., 2000). Additionally, liposomes attained close proximity to bacteria because of their penetration through the mucus layer (T. Yu et al., 2015). This had led to the approval of liposomal antibiotic formulations, i.e. Doxil and AmBisome by FDA (Barenholz, 2012; Boswell et al., 1998). In particular, ARIKAYCE, a liposomal antibiotic formulation was approved by FDA for the treatment of *Mycobacterium avium* complex lung disease (O. Khan & Chaudary, 2020). Recently, the amikacin encapsulated liposomes demonstrated greater penetration in biofilms and significantly higher cellular uptake in macrophages than non-formulated amikacin (J. Zhang et al., 2018). Liposomal formulations are a suitable candidate for pulmonary drug delivery because they provide superior safety profiles, decreased macrophage clearance, and sustained release (El-Sherbiny et al., 2011; Cipolla et al., 2014; Zhou et al., 2015). Wang and his coworkers successfully designed ciprofloxacin and colistin co-loaded liposomal formulations to evaluate the synergistic effects of antibiotics. Liposomal formulation with the size of 100 nm gave a sustained release. The *in-vitro* cytotoxicity studies showed efficient and safe antibacterial activity against clinical strains of *P. aeruginosa* and A459 (human epithelial cell lines) as compared to monotherapies (S. Wang et al., 2018). In another study, fucoidan-coated liposomes loaded with usnic acid were designed to target *M. tuberculosis*-infected macrophages (H37Ra). The liposomal vesicle size was 168 nm −1.18 µm in diameter and −5.41 mV zeta potential. The *in-vitro* cytotoxicity analysis revealed higher cellular uptake and lower IC_50_ values against infected macrophages as compared to uncoated liposomes (Lima Salviano et al., 2021). *A. baumannii* is a severe pathogen with a higher mortality rate and has clinical challenges due to limited therapeutic options. Liposomal thymoquinone was prepared, characterized and toxicity was evaluated by analyzing hematological, liver and kidney functional parameters against resistant *A. baumannii*. The *in-vivo* results revealed higher survival rate and reduced the bacterial load in lung tissues, inflammation markers, leukocytes, and neutrophils in blood of infected mice in compasrion with controls (Allemailem et al., 2021). Some recent liposomal formulations are described herein in Table 1.

**Table 1.** Liposomes as a drug delivery system for the infectious respiratory tract.

Nanosystems	Experimental model	Drug	Characteristics	Outcomes	Ref
Liposomes	BCI-NS1.1 cell lines	Curcumin	271.3 ± 3.06 nm; −61.0 ± 0.68 mV	Enhanced anti-inflammatory effects on lipopolysaccharides-induced airway inflammation	(Z. Y. Ng et al., 2018)
	*P.aerugeinosa strains*	Cefoperazone	410.85 ± 26 nm	Increased antibacterial activity; biofilm inhibition	(Ghodake et al., 2020)
	Murine pulmonary *P.aerugeinosa* infection	Colistin	118-136 nm	Sustained bactericidal activity; increased survival of mice	(Y. Li et al., 2017)
	BEAS-2B/mice	N.D	173.23 ± 1.62 nm; −0.82 ± 0.24 mV	Decreased airway hyper-responsiveness and pro-inflammatory cytokines	(Komalla et al., 2020)
	*P.aerugeinosa strains and* Calu-3 cell lines	Ciprofloxacin + Colistin	N.D	Improved antimicrobial activity; enhanced mucus membrane interaction	(Chai et al., 2019)
	Murine model of *M. tuberculosis* H37Rv infection	Licorice extract	210 nm; −32 to −28 mV	Decreased bacterial count in lung & sleep of TB infected mice	(Viswanathan et al., 2019)
	Murine model of *S. aureus*	Levofloxacin	200- 300 nm	Infiltration of inflammatory cells; effective anti-microbial and anti-biofilm activity	(Gupta et al., 2018)
	Clinical strains of *P. aeruginosa*	Levofloxacin	160 nm; −7.9 mV	Increased antibacterial activity due to the improved interaction with bacterial membrane	(Derbali et al., 2019)
	Asthma mice	Bergenin	158.33 ± 5.88 nm; 24.51 ± 0.51 mV	Inhibited inflammation; improved histopathological conditions	(X. Yu et al., 2021)
	Rats infected with *P.aerugeinosa*	Tobramycin	907.3 ± 40.1 nm	Reduction in the bacterial count; enhanced anti-inflammatory and anti-microbial efficacy	(Alhariri & Omri, 2013)

*BCI-NS1.1 cell lines: Primary airway basal cells; BEAS-2B: human non-tumorigenic lung epithelial cell lines; Calu-3 cell lines: non-small-cell lung cancer cell lines; N.D: Not determined*.

### 4.3. Solid lipid nanoparticles (SLN)

SLN is another physiological lipid-based nanoscale aqueous suspension comprised of triglycerides and phospholipids which are structurally different from liposomes. SLN offers a variety of advantages over traditional drug delivery systems such as increased drug loading, high drug stability, sustained and prolonged release, encapsulation of both hydrophilic and hydrophobic drugs, avoidance of organic solvent in formulation, low carrier toxicity, large-scale production, and site-specific drug delivery (Bi et al., 2009; Üner & Yener, 2007; Weber et al., 2014). Due to the physiological components and less toxicity, SLN formulations are more acceptable for pulmonary delivery (Table 2). Phospholipids are omnipresent in deep areas of the lungs and are important for the functioning of breathing. At the alveolar surface, phospholipid-based surfactant protein is available which maintains the optimal surface tension and reduces friction in the lung tissues (Beck-Broichsitter et al., 2011). The toxicity of SLN in *in-vitro* and *in-vivo* lung models showed SLN20 (20% phospholipids in lipid matrix) could be a safe pulmonary drug delivery carrier (M. Nassimi et al., 2010; Nassimi et al., 2009). Recently, the chitosan-coated SLN loaded with rifampicin was formulated which showed higher loading capacity, enhanced mucoadhesive property, and efficient permeability in alveolar epithelial cell A549 (Vieira et al., 2018). In another study, Pastro and coworkers developed sodium colistimethate-loaded SLN to evaluate the antibacterial activity against *P. aeruginosa* and to assess the *in-vivo* pulmonary distribution. The results revealed higher antimicrobial activity against clinically isolated *P. aeruginosa*. The *in-vivo* distribution showed the homogeneous spread of nanoparticles through the lung with no migration of SLN to other organs (Pastor et al., 2014). The targeting efficacy of SLN can be improved via different modifications which thereby increase the drug concentration at a specific site and decrease toxicity. Mannose modified SLN increased the absorption capacity of macrophages of encapsulated drug and enhanced mycobacterial activity against murine macrophage cell lines (Maretti et al., 2019).

**Table 2.** SLN as drug delivery systems for the infectious respiratory tract.

Nanosystems	Experimental model	Drug	Characteristics	Outcomes	Ref
Solid Lipid nanoparticles	J774 murine cell lines	Rifampicin	400 ± 20 nm; −35.33 ± 0.29 mV	Enhanced absorption of macrophages	(Maretti et al., 2019)
	RAW 264.7 cell lines	Isoniazid, Rifampicin and pyrazinamide	162.7 − 373.6 nm; −34.5 to −48.6 mV	Improved penetration of nanoparticles; increased antibacterial activity	(Khatak et al., 2020)
	RAW A549 and 264.7 cell lines, Wistar rats	Isoniazid	236 ± 9 nm; −19 ± 2 mV	Infiltration of inflammation cells; higher antibiotic efficacy; improved macrophage aggregation	(Ma et al., 2021)
	Female Swiss mice	Rifampicin	480- 850 nm; −8.5 to 55 mV	Increased anti-inflammatory response; higher drug retention in the pulmonary region; efficient alveolar macrophage phagocytosis	(Truzzi et al., 2020)
	H441 cell lines; mice	Grape seed-derived proanthocyanidins	243 ± 24 nm; −14.5 ± 1.0 mV	Reduced oxidative stress; decreased the inflammation of airway epithelial cells	(Castellani et al., 2018)
	A549 cell lines	Rifampicin	245- 344 nm; +40 mV	Higher mucoadhesive property; efficiently permeability to alveolar epithelial cells	(Vieira et al., 2018)
	Strains of *E.coli*, *P. aeruginosa*, *E. faecalis* and *S. aureus*	Ciprofloxacin	315 − 345 nm; 35.1 ± 0.81 46.1 ± 0.46 mV	Increased antibacterial activity; better interaction and penetration through a bacterial cell wall	(Pignatello et al., 2018)

*J774 cell lines: Murine macrophage cell lines; RAW 264.7 cell lines: Murine macrophage cell line; RAW A549: lung carcinoma epithelial cell lines; H441: Airway epithelial cell lines*.

### 4.4. Polymeric nanoparticles (PNPs)

A polymer is a group of large molecules comprising various small homogenous molecules. Polymers are classified into natural (chitosan, albumin, cyclodextrin, gelatin, collagen, and alginate) and synthetic [poly-lactic-co-glycolic acid (PLGA); polyethylene glycol (PEG), polyacrylates, poly-anhydrides, and poly-ethyleneimine (Rytting et al., 2008). Polymers offer feasible encapsulation strategies for the drug in different forms, i.e. microparticles, nanoparticles, and nano-embedded microparticles (Sung et al., 2007). The small size of PNPs allows the capillary penetration and uptake by cells which ultimately increases concentration at a specific site (L. Singh et al., 2017). PNPs have numerous advantages such as excellent biocompatibility, efficient targeting ability, stability, surface fabrication ability, protection of the drug from degradation, high encapsulation of drug, long shelf life, and sustained drug release (Menon et al., 2014). PNPs can deliver different agents which are incorporated into the surface of the polymer or dispersed in a polymeric matrix (Marasini et al., 2017). Polymers are considered a suitable candidate for pulmonary delivery. A variety of polymers are used to treat different bacterial respiratory infections, some of them are summarized in Table 3. HPOX (hydroxy benzyl alcohol incorporated polyoxalate) is a biodegradable polymeric prodrug of hydroxy benzyl (HBA) and a drug carrier that incorporates HBA and peroxalate through an ester linkage. HPOX suppresses the generation of ROS and has antioxidant and anti-inflammatory properties (Park et al., 2010). Recently, Yoo et al., prepared HPOX nanoparticles to evaluate their potency as a therapeutic agent for airway inflammatory diseases *in-vitro* and *in-vivo*. The nanoparticles were administered through the intratracheal route. The *in-vitro* analysis showed a decrease in the level of pro-inflammatory cytokines and ROS. The HPOX nanoparticles significantly reduced recruitment of pro-inflammatory cells in asthma allergic mice. Thus, these polymeric nanoparticles exhibit tremendous ability as therapeutics to treat airway inflammation and asthma (Yoo et al., 2013). PLGA is one of FDA approved co-polymer for therapeutic use in humans in numerous drug delivery systems because of its superior biocompatibility, biodegradability profile, and safety (Zhao et al., 2014). PLGA nanoparticle fabricated with tufstin (a natural immunostimulatory tetra-peptide with macrophage targeting and stimulatory property) derivative demonstrated enhanced internalization rate and intracellular activity of encapsulated drug against *M. tuberculosis* (Horváti et al., 2018). Günday Türeli et al., formulated ciprofloxacin-loaded PLGA nanoparticles to test the therapeutic effects on bacterial infection-induced CF in Calu-3 cell lines. The nanoparticles gave effective drug loading and permeability. Additionally, the low drug dose decreased the side effects in both *in-vitro* and *in-vivo* studies. The hydrophobic nature and nonspecific interaction of PLGA hinder their diffusion in mucus and bacterial biofilm. The surface modification of PNPs with PEG showed a high degree of free movement in biofilm in comparison with drug and lipophilic molecules (Forier et al., 2013; Sigurdsson et al., 2013). PEGylated nanoparticles can transport antimicrobial agents in biofilms of *P. aueroginosa*, *Burkolderia cepecia,* and *Burkholderia multivorans* (Forier et al., 2013; Messiaen et al., 2013). Another polymer that is in demand for its antimicrobial and mucoadhesive properties is chitosan. Chitosan is a natural, biocompatible polysaccharide with low immunogenicity and nontoxic nature. Recently, *in-vitro* studies of clofazimine-loaded chitosan nanoparticles demonstrated enhanced anti-mycobacterial activity, internalization, and bio-adhesion against C2C12 and H37Rv (a standard strain of *M. tuberculosis*) (Pawde et al., 2020).

**Table 3.** PNP as drug delivery systems for the infectious respiratory tract.

Nanosystems	Experimental model	Drug	Characteristics	Outcomes	Ref
Chitosan/ alginate	*P. aeruginosa* strains	Tobramycin	458 ± 31.1 nm; −19.2 ± 2.1 mV	Inhibited anti-inflammatory response; enhanced interaction with cystic fibrosis mucus; improved antimicrobial effects	(Hill et al., 2019)
PLGA	Calu-3 cell lines	Ciprofloxacin	190.4 ± 28.6 nm	Increased penetration of mucus and biofilm; stability in mucus; greater antibacterial activity	(Günday Türeli et al., 2017)
PLGA	*P. aeruginosa* strains; CFBE410; Calu-3 cells;	Ciprofloxacin	236.7 ± 22.6 nm	Enhanced antimicrobial activity; reduced biofilm fraction	(Juntke et al., 2021)
Chitosan	*P. aeruginosa* strains; wistar rats	Ciprofloxacin	212.3 ± 8.9 nm; −14.6 ± 1.3 mV	Prolonged microbial inhibition; prevented biofilm development	(Patel et al., 2020)
Chitosan coated PLGA	*P. aeruginosa* strains	Tobramycin	220.7 to 575.77 nm; +33.47 to 50.13 mV	Enhanced Antimicrobial activity and mucoadhesive properties with sustained release	(Al-Nemrawi et al., 2018)
Chitosan	H37Rv; C2C12 cell lines	Clofazimine	132-184 nm;	Higher cellular uptake across the mycobacterial membrane; increased mycobacterial activity	(Pawde et al., 2020)
Chitosan	H37Rv cell lines	Rifampicin	130-140 nm; 38.5 mV	Preferential nanoparticles uptake by macrophages; promising bactericidal action	(Prabhu et al., 2021)
PLGA	*P. aeruginosa* strains	Azithromycin	92 nm; −27 mV	Improved antimicrobial activity; biofilm prevention	(F. Wan et al., 2019)
PLA/PEG	16 HBE cell lines	Ibuprofen	<200 nm; −7.8 to −3.6	Enhanced anti-inflammatory effect; improved mucus penetration	(Craparo et al., 2016)
PLGA	*P. aeruginosa* strain*;* THP-1	Curcumin	105 ± 1.5 nm; −9.1 ± 4.6 mV	Decreased inflammatory cytokines; enhanced penetration in mucus; increased antibiotic activity	(Lababidi et al., 2020)
Chitosan	*Clinical isolates of A. baumannii*	Colistin,; meropenem; tigecycline;	+37.7 mV	Enhanced inhibitory; antimicrobial activity; suspectibility of MDR strains of *A. baumannii*	(Banoub et al., 2021)

*Calu-3 cell lines: non-small-cell lung cancer cell lines; CFBE410: human CF bronchial epithelial cell lines; H37Rv: M. Tuberculosis standard strain; 16 HBE cell lines: human bronchial epithelial cell lines; THP-1: immortalized monocyte like cell lines*.

### 4.5. Dendrimers

Dendrimers are highly branched three-dimensional polymeric nanostructures that are different from traditional polymers. Dendrimers have been used as suitable nanosystems due to internal cavity and surface modification. The functional groups are decorated on the surface of dendrimers through electrostatic interactions to enhance biocompatibility and versatility (D. Huang & Wu, 2018; Mehta et al., 2019). The drug can be encapsulated in interval cavities or conjugated to the surface of the dendrimer. Additionally, the dendrimers with amphiphilic characteristics can deliver drugs with different solubilities (Ahmad et al., 2015). Several types of dendrimers have been described based on dendrimers’ synthesis, shape, physicochemical properties, and structures (Filipczak et al., 2021). Polyamidoamine (PAMAM) and polypropylene amine are the most common and oldest dendrimers which are used in drug delivery to their efficient solubilizing ability, reduce toxicity, and enhanced biocompatibility (Idris et al., 2020; Kretzmann et al., 2017). Recently, PAMAM dendrimers were used for the delivery of siRNA to treat chronic lung inflammation. The siRNA delivery was evaluated *in-vitro* using RAW264.7 macrophage cell lines and *in-vivo* through the murine model of lipopolysaccharide-induced lung inflammation. The PAMAM/siRNA nanosystem showed increased cellular uptake in macrophages while *in-vivo* studies revealed tumor necrosis factor α inhibition upon administration (Bohr et al., 2020). Another study reported the inhalable nano drug-using rifampicin-loaded PAMAM dendrimers for the treatment of tuberculosis. The PAMAM dendrimers gave controlled release, improved drug absorption, and bioavailability in comparison with intravenous administration (Rajabnezhad et al., 2016). Some recent advancements in dendrimers are explained in Table 4.

**Table 4.** Applications of dendrimers as drug delivery systems for the infectious respiratory tract.

Nanosystems	Experimental model	Drug	Characteristics	Outcomes	Ref
Dendrimer	*P. aeruginosa; G. mellonella* larvae	N.D	N.D	Prevent biofilm formation; enhanced antimicrobial effects	(Pompilio et al., 2018)
	N.D	Rifampicin	2.93 ± 0.02 nm	Increased stability; rapid pH-dependent release	(Bellini et al., 2015)
	H37Ra	Isoniazid	922 ± 21.77 nm; 4.55 ± 2.3 mV	Higher drug loading capacity; controlled drug release rate; inhibit bacterial activity	(Rodrigues & Shende, 2020)
	Wistar rats	Rifampicin	6.21 ± 0.03 nm	Enhanced bioavailability and absorption; controlled release	(Rajabnezhad et al., 2016)
	RAW 264.7 cell lines	Rifampicin	>12 mV	Greater loading potential; Prolonged and sustained drug release; no toxicity; higher interaction with cell membrane; the greater survival rate	(Ahmed et al., 2021)
	Lung metastatic mouse model	Doxorubicin	4.7 to 9.7 nm; 13.8 ± 7.0 mV	Decreased tumor rate; the increased survival rate of mice; enhanced accumulation, efficacy & retention time of drug	(Zhong et al., 2016)
	H37Rv; H37Ra; BCG; BALB/c mice	Rifampicin; Isoniazid; delmanid	N.D	Higher anti-mycobacterial activity; reduced bacterial count; increased survival rate	(Mignani et al., 2021)

*H37Rv/H37Ra: M. Tuberculosis strain; RAW 264.7 cell lines: Murine macrophage cell line; BCG: Bacillus Calmette-Geurin vaccine for tuberculosis; N.D: Not determined*.

PNPs have gained considerable progress so far due to the ease of their penetration through the mucus layer and gave efficient therapeutic effects even at a very low dose. However, some polymers, i.e. chitosan have shown biodegradability concerns in *in-vivo* administration. Polymer-based nanosystems may also demonstrate a sustained release, although the drug release mechanism is still unclear and under scrutiny. Thus, *in-vivo* optimization is required to evaluate the safety of nanoparticulate.

### 4.6. Nanogels

Hydrogels are the meshed network of polymers that can absorb high water content. The term nanogel is used for hydrogels that lie in the nano scale range. Thus the nanogels possess dual properties of nanoparticles and hydrogels. Due to their small size, the nanogels not only interact with the cell but also penetrate inside the cell. The network of nanogels provides space for the integration of drugs inside them (Keskin et al., 2021). Nanogels possess a higher loading capacity of the drug (more than 30% of their weight) and are one of the best candidates for the controlled, stimuli-responsive release of the drug. By changing the chemical composition of the nanogels the desired characteristics (size, charge, porosity, softness) can be achieved (Soni et al., 2016). The surface of nanogels can be engineered for ligand attachment and their higher mechanical strength contributes to their higher stability (Hamzah et al., 2017). The properties like high loading capacity, improved stability, larger surface area for bioconjugation, tunable size, and responsiveness to environmental stimuli make the nanogels promising carriers for drug delivery. Kabanov & Vinogradov (2009) formulated a nanogel for *S. pneumonia* which is involved in respiratory tract infections. The cholesteryl-group-containing pullulan (CHP) was assembled into a spherical shape nanogel through hydrophobic interactions. Pneumococcal surface proteins A (PspA) antigen is encapsulated inside this pullulan nanogel and the pneumococcal surface proteins A (PspA) antigen incorporated CHP nanogel (CHP-PspA) was developed as a vaccine against *S. pneumoniae.* The administration of PspA-loaded nanogel to mice through the pulmonary route exhibited more promising results than the administration of PspA antigen alone. The nanogel inhibited the growth of *S. pneumoniae* in the nasal cavity and lungs of mice. The clinical trials of this nanogel on non-human primates are delivering encouraging results, thus opening new avenues for the better treatment of respiratory tract infections (Aderibigbe & Naki, 2018; Fukuyama et al., 2015; Gyu Kong et al., 2013; Nakahashi-Ouchida et al., 2018).

### 4.7. Metal nanoparticles

Metal nanoparticles have gained attention due to their surface energy, greater surface-to-volume ratio, spatial confinement, and negligible imperfections. Metal nanoparticles have distinctive physicochemical, thermal, electrical, optical, mechanical, thermal, and biological properties. Biosythesized noble nanoparticles (gold and silver nanoparticles) have emerged as potential weapons in the antibacterial arsenal because of their antimicrobial efficacy Noble nanoparticles have also shown tremendous biomedical applications for treating a wide range of diseases (Ahangarpour et al., 2018; Ameen et al., 2018; Sathishkumar et al., 2016).

#### 4.7.1. Silver nanoparticles (AgNPs)

AgNPs are defined as nanomaterials with all dimensions ranging between 1 and 100 nm. AgNPs exhibit unique electrical, catalytic, and optical properties which have led to the investigation and modification of products for imaging (Kumar et al., 2018), diagnosis (Hasanzadeh et al., 2019), targeted drug delivery (Kooti et al., 2018), detection and antimicrobial activity (Roh et al., 2019). Silver is an excellent antibacterial agent, nontoxic inorganic metal, and nontoxic to human cells with a concentration limit of 350 µg/day. The AgNPs have been proved to be a promising antimicrobial agent to combat *in-vitro* and *in-vivo* bacterial infections (Bruna et al., 2021). AgNPs showed efficient antibacterial activity to a wide range of microorganisms including *E.coli* (W. R. Li et al., 2010), *Candida albicans* (K. J. Kim et al., 2009), *P. aeruginosa* (Kora & Arunachalam, 2011), and *S. aureus* (W. R. Li et al., 2011). and The combined action of antibiotics and AgNPs has proved capable of killing microorganisms via different mechanisms. These mechanisms include; direct contact with bacterial components, i.e. biofilms and cell wall; generation of reactive oxygen species (ROS); inhibition of bacterial DNA replication; release of bioactive ions (Ag^+^); alteration of cell wall and cytoplasm; disruption of metabolic pathways. The bactericidal effect of AgNPs depends on nanoparticle size, shape, surface area, surface modification, and rate of Ag^+^ generation, structural differences in gram-positive and gram-negative bacteria (Tăbăran et al., 2020). Recently, AgNPs gave remarkable antibacterial activity in comparison with tobramycin (antibiotic) against bacterial strains. Pompilio et al., have formulated novel AgNPs against *P. aeruginosa, S. maltophilia, S. aureus,* and *B. cepacia* recovered from patients with CF. The AgNPs *in-vitro* assayed showed a rapid bactericidal effect against *B. cepacia and P. aeruginosa.* The viability reduction, destruction of extracellular matrix, and cell death were also observed against *S. aureus* and *P. aeruginosa* biofilms (Pompilio et al., 2018). Biogenic AgNPs also exhibited promising *in-vitro* and *in-vivo* antibacterial activity against *S. aureus*. Biogenic AgNPs were synthesized from *Gardenia thailandica* leaf extract followed by their characterization. The characterized nanoparticles were spherical with 11.02 − 17.92 nm diameter. The antibacterial activity of AgNPs was evaluated *in-vitro* and *in-vivo* against *P. aeruginosa* clinical isolates. The *in-vitro* flow cytometry demonstrated that AgNPs reduced the membrane potential and also decreased the membrane integrity. The *in-vivo* examination was done on *S. aureus* infected wounds in rats. The AgNPs treated rats resulted in epidermis regeneration and reduced infiltration of inflammatory cells (Attallah et al., 2022). Respiratory tract microbes infections can also be treated through biogenic AgNPs in combination with antibiotics. Aremu et al., developed AgNPs from *Hypoxis hemerocallidea* (Southern African plant) to evulate antibacterial activity against *S. pneumonia*, *P. aeruginosa, Bacillus cereus, E. coli,* and *M. catarrhalis.* Broad spectrum of antibacterial activity was observed against all respiratory pathobionts. However, AgNPs also synergistically increased the antibacterial effect of streptomycin when they were used in combination (Aremu et al., 2021). Similarly, AgNPs were combined with antibiotics (polymixinB, rifampicin and tigecycline) against resistant *A. baumannii* clinically isolated from patients. The AgNPs showed significant antibacterial activity with no cytotoxicity against A549 and HL-7702 cells. *In-vivo* studies increased the survival rate and decreased the level of proinflammatory cytokines, interlukins and tumor nacrosis factor alpha (TNF-α) in mice (G. Wan et al., 2016). Therefore, AgNPs have dual function which can synergistically enhance activity and also act as a carrier for small molecules.

#### 4.7.2. Gold nanoparticles (AuNPs)

AuNPs are widely used in biochemical and pharmacological research owing to their chemically inert character, ease of penetration, the possibility of being functionalized with different molecules, biocompatibility, controlled release, low toxicity, and higher stability among metallic nanoparticles (de Menezes et al., 2021; Dykman & Khlebtsov, 2012; Y. Zhang et al., 2015). Targeted molecular imaging (Lan et al., 2013), biosensing (Kao et al., 2013), and targeted drug delivery (Hema et al., 2018) can also be achieved through AuNPs. AuNPs are highly explored and used as therapeutic agents against microbial infections (F. Khan et al., 2019; Rajkumari et al., 2017; Rice et al., 2019). Recently, Alsamhary et al., used tricetin to synthesize AuNPs to evaluate the *in-vitro* antibacterial efficacy against bacterial pathogens which were isolated from immunocompromised patients suffering from respiratory infections. The antibacterial studies confirmed the broad-spectrum antimicrobial activity of AuNPs against *S. aureus*, *Acinetobacter pittii*, *P. aeruginosa*, *Enterobacter xiangfangensis*, *Proteus mirabilis*, *Bacillus licheniformis*, *Aeromonas enteropelogenes*, and *Escherichia fergusonii.* The *in-vitro* cytotoxicity results revealed that biosynthesized AuNPs were biocompatible on primary normal human dermal fibroblast cells up to 50 µg/mL (Alsamhary et al., 2020). In another study, folic acid-decorated docetaxel-loaded AuNPs were synthesized to evaluate anti-cancer activities by *in-vitro* studies against lung cancer cell lines (H520). The cytotoxicity analysis revealed a 50% reduction in cell viability in comparison with control due to the combined effect of AuNPs-based nanoconjugates (Thambiraj et al., 2019). Water-soluble and highly stable chitosan oligosaccharide capped gold nanoparticles formulation (COS-AuNPs) was designed to inhibit the phenotypic traits (biofilm formation, virulence factors production), and motility of *P. aeruginosa.* COS-AuNPs inhibited the formation of biofilm and eradicated the preexisted mature biofilm. COS-AuNPs also hindered the bacterial hemolysis and reduced the production of some virulence factors from *P. aeruginosa.* Attenuation of bacterial swimming and twitching motilities were observed during the treatment of COS-AuNPs. However, an efficacy test using an animal model is required that will confirm that COS-AuNPs can be used as a potential agent to control the infections associated with *P. aeruginosa* (F. Khan et al., 2019). Thus, AuNPs-based nanoconjugates can be considered an alternative and promising carrier for the treatment of respiratory infections.

Most of the metallic nanoparticles were effective in *in-vitro* antibacterial activity such as bacterial toxic cations release or ROS but these mechanisms were diminished by numerous *in-vivo* considerations. Metal cations might be attracted by host molecules and can deviate nanoparticles from antibacterial activity while ROS produced by nanoparticles could be decreased or neutralized in *in-vivo* from biomolecules (uric acid, albumin, ascorbic acid, glutathione). Furthermore, biodistribution and excretion kinetics have to be studied in detail for different animal models because metallic nanoparticles are not biodegradable. The excretion of metallic nanoparticles from the liver and spleen can take up to 3–4 months that possibly can induce inflammation. Aggregated nanoparticles can exhibit catalytic activity and interfere with numerous diagnostic techniques. Above mentioned concerns, along with toxicity are the challenges of metallic nanoparticles in clinical applications.

## 5 Mitochondrial STAT 3 and the role of nanocarriers

Signal transducer and activator of transcription 3 (STAT 3) is a transcription factor that functions either through the classical pathway or non-classical pathway. In the classical pathway, it is translocated to the nucleus where it mediates gene transcription while in the non-classical pathway it is translocated to the mitochondria (Yang & Rincon, 2016). STAT 3 causes effective regeneration of NAD + in respiration by interacting with the complex I in mitochondria which leads to an increased dehydrogenase activity of Nicotinamide Adenine Dinucleotide (NADH). The NAD + results in the induction of antioxidant genes as it acts as a retrograde signal (Lahiri et al., 2021). Fu et al. (2022) demonstrated that mitochondrial STAT 3 plays a crucial role in the functioning of innate lymphoid cells (ILC2) that are involved in type 2 immune response. STAT 3 enhances the ILC2-led allergic inflammation in the lungs. However, decreasing the STAT3 levels and inhibiting the translocation of STAT3 to mitochondria has reduced the inflammatory allergic responses of ILC2. Das et al. (2014) formulated Polyethylenimine (PEI) and PLGA nanoparticles for the encapsulation of siRNA to inhibit the STAT 3. The nano-encapsulated siRNA exhibited effective silencing of STAT 3 in A549 cells while the unencapsulated siRNA administration did not deliver promising results. This indicates the effectiveness of nanocarriers in lung cancer therapy.

## 6. Detection through nanodiagnostics

Infectious diseases are the primary cause of death in developing countries with more than 95% of deaths related to infectious diseases being caused because of the failure of effective diagnosis and treatment strategies. Approaches like culture and microscopy, immunology, and PCR are conventionally utilized for the detection of pathogens. However, these conventional detection methods are inefficient for the early and rapid detection of pathogens as these are time-consuming (e.g. culture and microscopy method), expensive, sophisticated, and also carry the risk of cross-contamination. Thus, to overcome the challenges of the conventional diagnostic techniques, nanoparticles and nanodevices-based diagnosis could be carried out for infectious pathogens. Nano diagnostics are robust, cost-effective, portable, and user-friendly (Noah & Ndangili, 2019). The high surface area of nanoparticles makes the nanodiagnostics more sensitive and robust(Prasad, 2014). *Currently,* nanomaterials like gold nanoparticles, silver nanoparticles, magnetic nanoparticles, carbon nanotubes, and quantum dots have been explored for their potential as diagnostic tools. Gold nanoparticles are more feasible for clinical diagnosis owing to their properties like biocompatibility, inertness, and unique optical features. Magnetic nanoparticles like iron oxide are extensively studied for their applications in biomedicine. They are employed to detect pathogens by functionalizing their surface with recognition molecules (antibodies, carbohydrates) (Homayoonnia et al., 2021; J. Kim et al., 2017). Magnetic nanomaterials like gold and silver nanoparticles exhibit Surface Plasmon Resonance and are used in place of labels to detect the pathogens upon hybridization of complementary bases the nanomaterial will change color (Lambe et al., 2016). Simple nanoparticle-based diagnosis could not deliver the anticipated results in the diagnosis of pathogens in complicated infections (e.g. where many strains of pathogens are involved) and thus calls for the need for more advanced diagnostic tools. So, nano-diagnostic devices are engineered through the integration of nanotechnology in many techniques (Y. Wang et al., 2017). These nanotechnology-based diagnostic assays are also known as pen side tests and lab on chip diagnostic tests as they surpass the need for sophisticated labs and skilled personnel (Lambe et al., 2016). DNA-based technologies for the analytical process like Microarray, rolling circle amplification, etc. utilize nanoparticles to aid the process of diagnosis (Shi et al., 2014). *Shi et al.* investigated the respiratory tract infection-causing bacteria *S. pneumonia* by using the rolling cycle amplification of DNA and utilizing gold nanoparticles as sensors (Salieb-Beugelaar & Hunziker, 2015; Shi et al., 2014).

## 7 Pre-clinical and clinical strategies

Various nanotechnology-based oral delivery systems have been formulated for the delivery of antibiotics and tested on animal models. The anti TB drugs such as rifampicin, pyrazinamide, and isoniazid-loaded SLN have been tested in rodents to improve their bioavailability (H. Singh et al., 2015). In infected mice, 46 daily doses of the free drug were administered orally to achieve the therapeutic effect. However, only five doses of SLN on every 10th day didn’t detect any tubercle bacilli (Pandey et al., 2005). Pulmonary administration of rifampicin-loaded SLN in rodents enabled delivery to alveolar macrophages (Chuan et al., 2013). Bacillus Calmette-Geurin (BCG), a TB vaccine that was approved in 1921 showed limited protection as compared to aerosolized formulation of BCG nano-microparticles. When this formulation was administered to guinea pigs, they produced elevated immune responses and resistance to TB infections than standard vaccines (Garcia-Contreras et al., 2008; Hawn et al., 2014; Kaufmann et al., 2017). Additionally, nano chitosan-based recombinant DNA vaccine enhanced immunologic and therapeutic effects in mice model against TB (Feng et al., 2013). Despite promising results in preclinical studies, only a few clinical trials have been conducted to test the nano-therapy in respiratory infections. A clinical trial has been completed to check the efficacy of Pitavastatin-loaded PLGA nanoparticles (Nakamura et al., 2017). Two liposomal formulations, i.e. amikacin and ciprofloxacin have reached the clinical stages for the treatment of CF-associated lung infection (Paranjpe & Müller-Goymann, 2014). However, no groundbreaking studies have been put forward in humans till now, and studies concerning treatment are still at the beginning that demands in-depth investigation.

## 8 Conclusion and future perspectives

**Figure 6.**
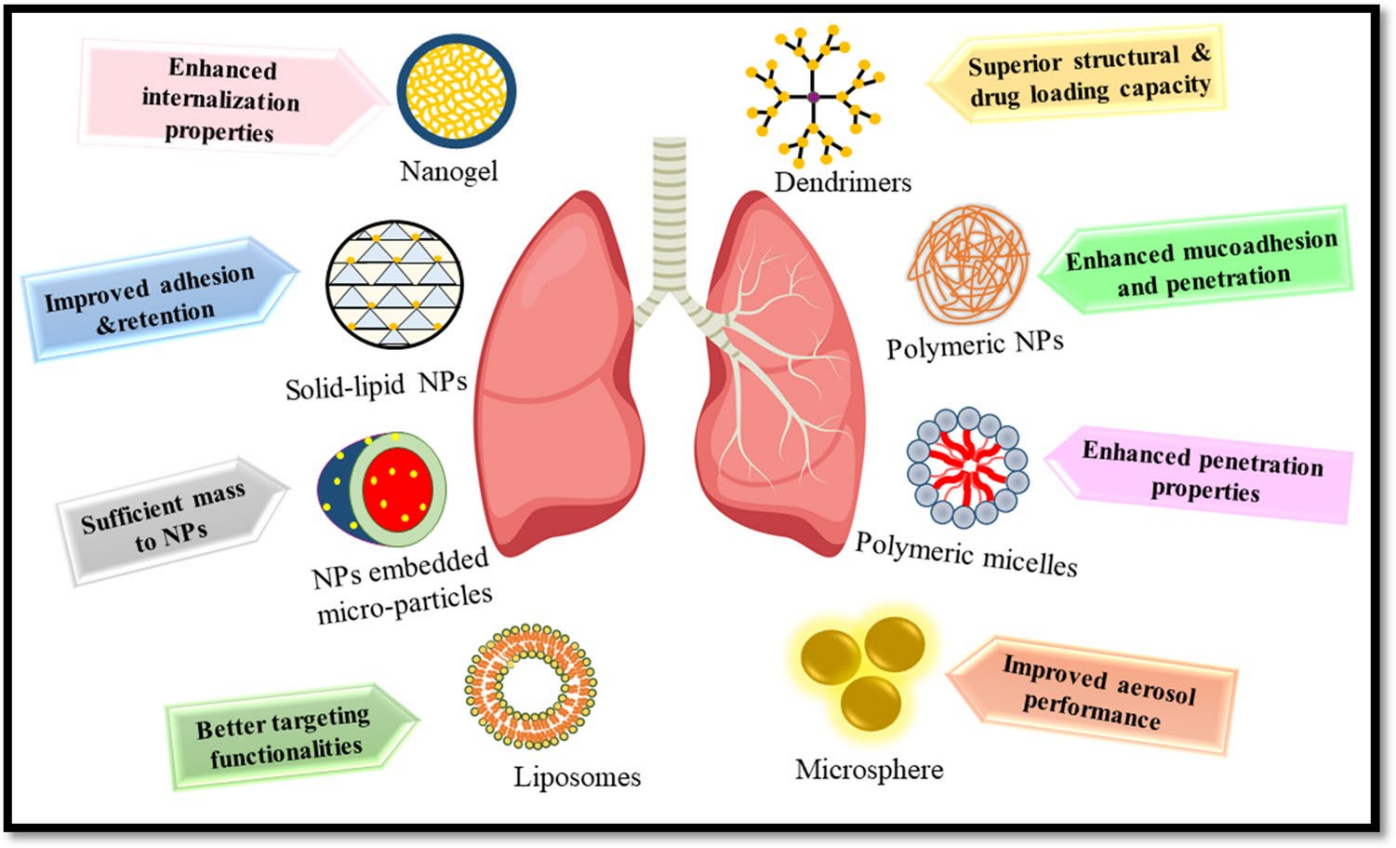
Overview of nano systems for respiratory tract infections.

Respiratory tract infections represent a myriad of challenges to the pharmaceutical industries and thus signal alternative approaches to combat the highly resistant and unaccessible pathogens lying deep inside the respiratory tract. Nanotechnology exhibits pronounced potential to overcome these challenges and nanomedicines have provided promising therapeutic effects (Figure 6). Although preclinical trials have shown broad development progress, clinical effects need to be verified. The toxicity and ultimate fate of nanoparticles in the body called for the rational design of nanoparticles. This manuscript focuses on the advantages and limitations of different nanosystems. Lipid-based colloidal systems (SLN and liposomes) have advantages over others due to the presence of physiological components in their formulations. Many liposomal formulations have been clinically approved by FDA, ARIKAYCE is one of the antibiotic formulations to treat infectious agent *M. avium. A. baumannii* is one of the challenging clinical microbes, thymoquinone liposomal formulation also gave promising *in-vivo* results against clinical isolates of this pathogen and the time is not far when we see a bunch of nanomedicines being approved after the clinical trials. Some other freeze-dried liposomal formulations have also reached clinical phase III studies. Future implications may involve targeted multi-functional drugs that will not only effectively target the bacterial cell but also suppress the resistance mechanism of bacteria. Such advanced multifunctional therapy will provide targeted delivery of drugs with a different mode of action; increase the efficacy of therapeutics to treat infected diseases and limit adverse effects on healthy tissues. Nanoantioxidant therapy is another approach that overcomes oxidative stress caused by free radicals and could display more promising results by limiting the side effects of antioxidants. However, for successful applications in therapeutics, more research needs to be conducted in the field of nanotoxicity and the ultimate fate of nanoantioxidant in the body. Valuable insight in this regard will be to evaluate the antioxidant potential of biopolymers as delivering these materials at nanoscale would result in enhanced bioavailability and less toxicity. The optimum dosage of nanoantioxidants is another avenue to be worked on. Furthermore, through nanotechnology new vistas of bacterial vaccines will open that can prevent the onset of bacterial respiratory infections.

## Data Availability

Not applicable.
